# Transcriptional control of strawberry ripening – two to tango

**DOI:** 10.1093/jxb/erx285

**Published:** 2017-09-30

**Authors:** Denise Tieman

**Affiliations:** University of Florida, Department of Horticultural Sciences, Gainsville, FL, USA

**Keywords:** DOF transcription factor, eugenol, *Fragaria*, fruit ripening, phenylpropanoid pathway, strawberry

## Abstract

This article comments on:

**Molina-Hidalgo FJ, Medina-Puche L, Cañete-Gomez CJ, *et al.*** 2017. The fruit-specific transcription factor FaDOF2 regulates the production of eugenol in ripe fruit receptacles. Journal of Experimental Botany **68,** 4529–4543.

**Although many genomic regions encoding putative transcription factors have been identified in sequencing efforts, the roles of relatively few have been elucidated. Now, working with strawberry, Molina-Hidalgo *et al.* (2017) have identified a second transcription factor (FaDOF2) involved in regulating eugenol synthase and production of the aroma compound eugenol during strawberry ripening. Not only is this important in understanding the control of ripening in non-climacteric fruit but, potentially, in breeding to reintroduce flavor into modern germplasm.**

Eugenol is a phenylpropanoid aroma compound that is an important constituent of the flavor of many spices, including cloves and basil (Koeduka *et al.*, 2006). It is also found in flowers and fruits, including banana, tomato, grape, melon and strawberry (Aubert and Pitrat, 2006; Tieman *et al.*, 2012), all of which are major agricultural species (Box 1). Eugenol synthase catalyzes the last step in the biochemical pathway to eugenol in strawberry (*Fragaria* × *ananassa*): the biosynthesis proceeds from coniferyl alcohol through the action of coniferyl alcohol acetyltransferase to form coniferyl acetate, the eugenol synthase substrate (Hoffmann *et al.*, 2011). Three forms of eugenol synthase have been identified in strawberry – FaEGS1a, FaEGS1b and FaEGS2 – with FaEGS2 being the dominant form in ripening receptacles (Aragüez *et al.*, 2013).

**Box 1. Strawberry cultivation and the aromatic, eugenol-containing crop**

**Figure:**
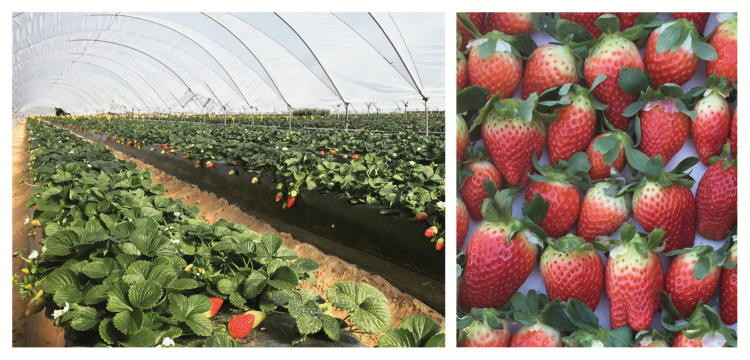

Images courtesy of Francisco Javier Molina-Hidalgo and Juan Muñoz-Blanco.

Earlier work showed that FaMYB10 (Medina-Puche *et al.*, 2014) works as a major regulator of the expression of genes belonging to the phenylpropanoid pathway, and that it is upstream of another MYB transcription factor, FaEOBII, also regulating its expression. In turn, FaEOBII regulates the expression of FaEGS2 (Medina-Puche, 2015). Molina-Hidalgo *et al.* (2017) have now identified a second transcription factor, FaDOF2, that regulates the levels of FaEGS2 transcription – silencing showed that it regulates the expression of FaEGS2, eugenol synthase and FaEOBII. FaDOF2 is expressed in red receptacles and flower petals, but not green receptacles; it is localized in the nucleus, binds to a defined DNA sequence found in the promoter of a strawberry eugenol synthase gene (FaEGS2) and interacts with the transcription factor FaEOBII. FaEOBII and FaDOF2 together coordinate the expression of FaEGS2.

Fruit ripening in climacteric fruits is regulated by the plant hormone ethylene, but regulation of ripening in non-climacteric species, such as strawberry, is not well understood. Nevertheless, several plant hormones, including ABA, auxins and gibberellins, have been implicated in the process (reviewed in Fortes *et al.*, 2015; Osorio *et al.*, 2013). While ABA increases during strawberry ripening, auxin and gibberellins decline, suggesting that ABA coordinates strawberry receptacle ripening (Medina-Puche *et al.*, 2015; Medina-Puche *et al.*, 2016). FaDOF2 expression is up-regulated during strawberry ripening and is regulated by ABA.

## Multiple changes in color, texture, taste and aroma

Fruit ripening is associated with multiple changes in color, texture, taste and aroma, and many genes exhibit changes in expression during the transition from development to ripening (Giovannoni *et al.*, 2017). These include genes encoding RNAs for cell wall-degrading enzymes, anthocyanin and carotenoid biosynthetic enzymes, chlorophyll-degrading enzymes, and enzymes in the pathways to flavor compounds (reviewed by Giovannoni, 2004, and Tohge and Fernie, 2015). Each fruit species has its own unique flavor, comprising a mixture of sugars, acids and aroma compounds. Although sugars and acids are the base of flavor, the particular mix of aroma compounds constitutes each fruit species’ unique signature. Altering the levels of these compounds in relation to one another can significantly alter the flavor of a fruit, resulting in reduced acceptability by consumers. Therefore, each species has to regulate the production of many biochemicals from multiple pathways to produce its own defining flavor (Wang *et al.*, 2016).

In addition, these aroma compounds are derived from many different primary metabolites, including carotenoids, amino acids, lipids and carbohydrates (reviewed by Schwab *et al.*, 2008). Moreover, each species must have multiple distinct pathways for the formation of aroma compounds, and many of these compounds are only produced in fruit, flowers or ripening fruit. In turn, each of these pathways must be regulated by transcription factors that coordinate the expression of the genes producing many different enzymes in a fruit- or ripening-specific manner. Considering that most fruits have hundreds of aroma compounds from many different pathways, the degree of complexity rapidly multiplies. Regulation of these pathways to produce these compounds during the relatively short fruit-ripening process requires transcriptional regulation of many genes and biochemical pathways simultaneously.

## Identifying the roles of transcription factors

The multiple complexities of the fruit-ripening process complicate the identification of the role of transcription factors in producing associated volatile compounds. Unlike enzymes involved in primary and secondary metabolism, sequence similarity among transcription factors does not mean that the regulated genes have similar functions (Klie *et al.*, 2014). Indeed DOF-family transcription factors have been shown to regulate a large number of processes: seed germination; the response to light; root and leaf development; responses to salicylic acid; nitrogen assimilation; carbon metabolism; organ growth; hormone signaling; and biosynthesis of glucosinolates, brassinosteroids, jasmonic acid and flavonoids (reviewed by Corrales *et al.*, 2017; Yanagisawa, 2004; Lehti-Shiu *et al.*, 2017). Although many genes encoding transcription factors have been identified through sequencing efforts in multiple plant species, relatively few of these have been assigned a function in controlling gene expression (reviewed by Karlova *et al.*, 2014; Kourmpetli and Drea, 2014; Tohge and Fernie, 2015). Next-generation sequencing will continue to identify transcription factors from more species that are up-regulated during ripening.

Considering the complex regulatory networks regulating processes such as fruit ripening, determining the targets of putative transcription factors and their interactions in ripening and flavor compound accumulation would further our understanding of the ripening process. The approach used by Molina-Hidalgo *et al.*, comparing transcriptomes of green and ripe strawberry receptacles, was effective in defining the role of FaDOF2 in the control of eugenol synthesis and it is a way forward in identifying transcription factors important for other ripening-related processes.

What about other implications? Loss of flavor in fruits and vegetables is a common consumer complaint. Breeding for yield, disease resistance, shelf-life, appearance and firmness for shipping has resulted in loss of flavor quality in modern fruit varieties. Although these traits are essential for modern production processes, it should be possible to reintroduce flavor into modern germplasm. With a greater understanding of the biochemical and regulatory processes in the formation of flavor compounds, breeding for improved flavor should be feasible. Some flavor biochemical pathways have been established, and are now targets for breeding for improved flavor (Tieman *et al.*, 2017). Transcription factors, such as FaDOF2, should only add to the target list.
